# Supplementary material for: Multi-scale mechanical characterization of highly swollen photo-activated collagen hydrogels
Source: J R Soc Interface. 2015 Jan 6;12(102):20141079. doi: 10.1098/rsif.2014.1079 (PMC4277102; doi:10.1098/rsif.2014.1079)
Supplement: Supporting Information [file rsif20141079supp1.doc]

Supporting Information

Title: Multi-scale mechanical characterization of highly swollen photo-activated collagen hydrogels

**Authors:** Giuseppe Tronci,^*^ Colin A. Grant, Neil H. Thomson, Stephen J. Russell, David J. Wood

^*^ Email: [g.tronci@leeds.ac.uk](mailto:g.tronci@leeds.ac.uk)


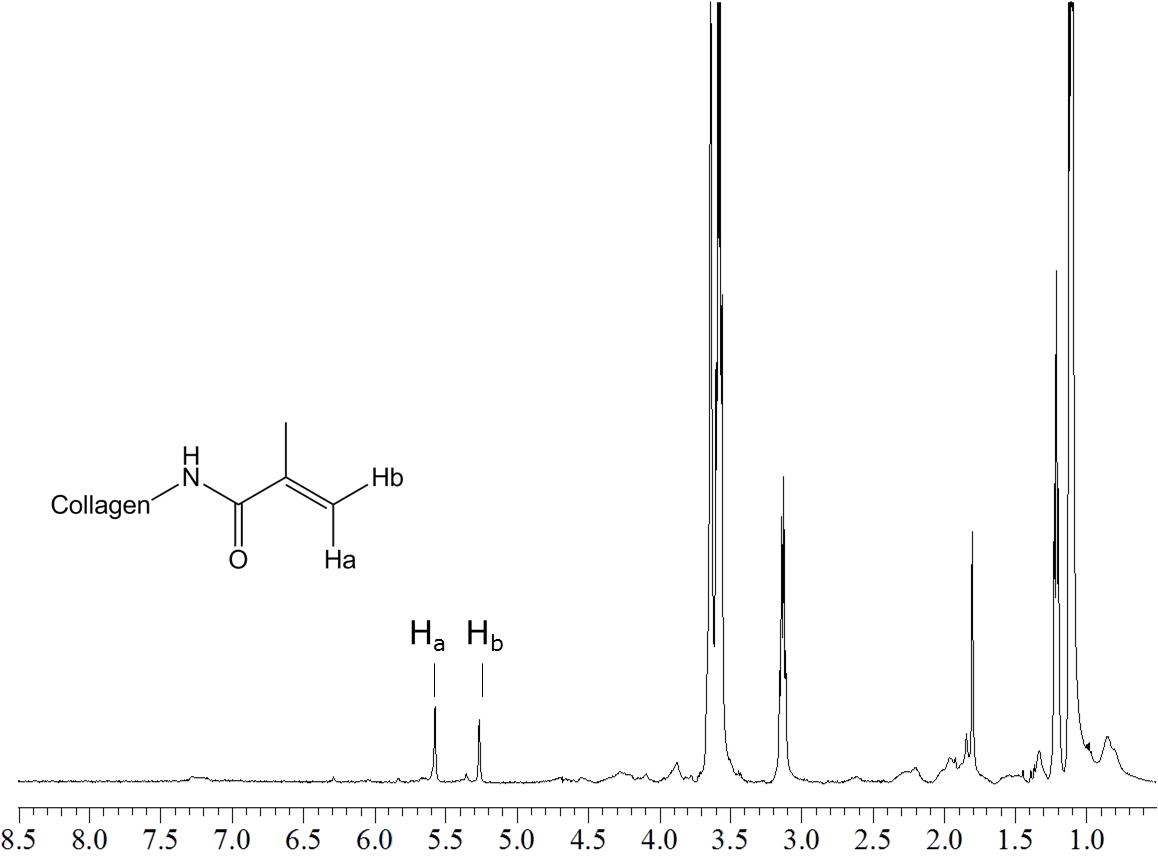


Figure S1. ^1^H-NMR spectra of sample CRT-MA25; additional peaks related to vinyl geminal protons of MA are depicted in the region 5.3−5.7 ppm.


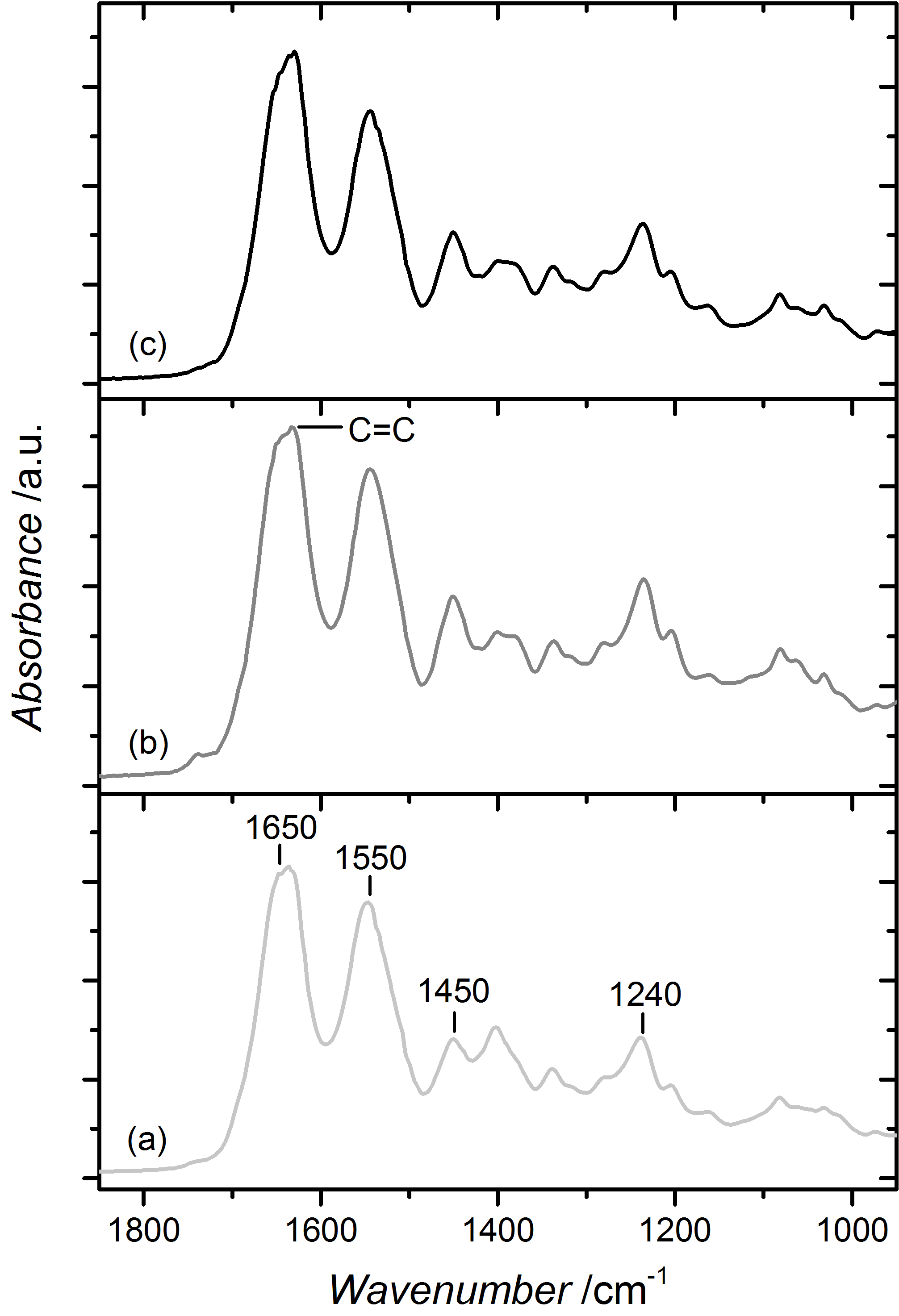


Figure S2. Exemplary ATR-FTIR spectra of CRT (a), CRT-GMA50 (b) and CRT-GMA50^*^ (c). An additional shoulder peak is observed at 1640 cm^-1^ in functionalized collagen (b), compared to native (a). Following UV irradiation, this peak is not clearly detected in the corresponding spectrum (c), providing evidence that a covalent network is present in the photo-activated sample.


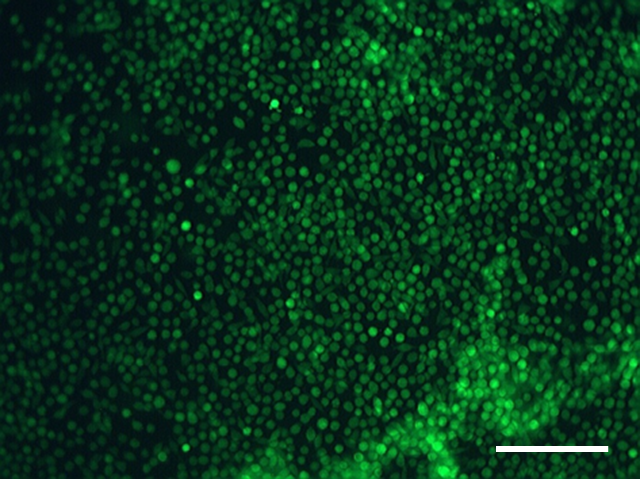

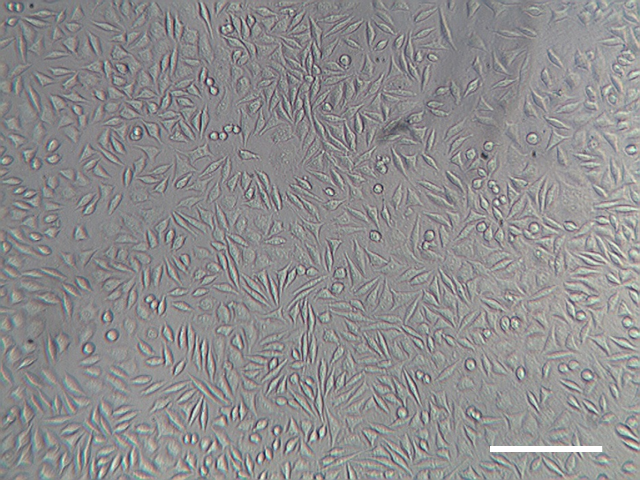


Figure S3. Left: 5-chloromethylfluorescein stained L929 mouse fibroblasts observed on a fluorescence microscope following 48-hour cell culture on hydrogel CRT-GMA50^*^. Right: Cell morphology of L929 mouse fibroblasts following 48‑hour cell culture on 72-hour extract of hydrogel CRT-GMA50^*^. Scale bar: 200 µm.


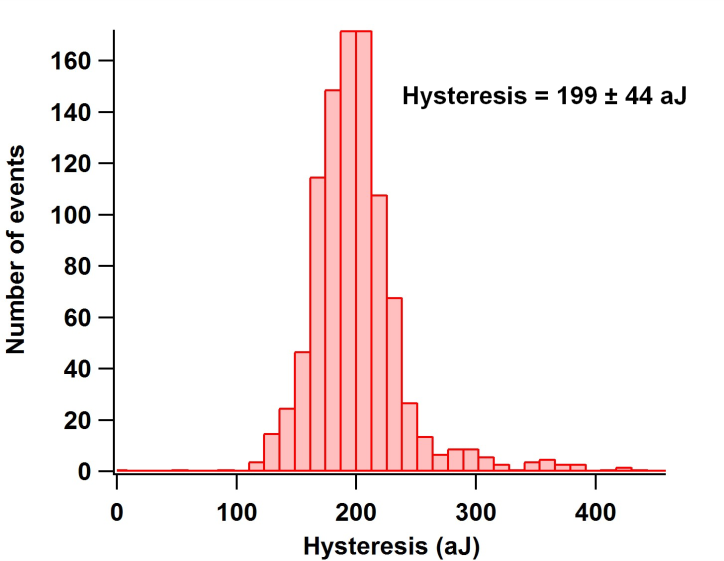


Figure S4. Hysteresis distribution in hydrogel CRT-MA10^*^ (n=1000 plots) obtained via AFM force mapping. Hysteresis values were determined from the area bounded between the loading and unloading curves throughout a force map.
